# Supplementary material for: Room-temperature polariton condensate in a quasi-2D hybrid perovskite
Source: Nat Commun. 2026 Jan 31;17:1261. doi: 10.1038/s41467-026-68723-7 (PMC12864779; doi:10.1038/s41467-026-68723-7)
Supplement: Supplementary file 1 — Supplementary Information [file 41467_2026_68723_MOESM1_ESM.pdf]

# Supporting Information:

# Room-temperature polariton condensate in a quasi-2D hybrid perovskite

Marti Struve[1], Christoph Bennenhei[1], Hamid Pashaei Adl[1], Kok Wee Song[2], Hangyong Shan[1], Nadiya Matukhno[1], Jens-Christian Drawer[1], Sven Stephan[3], Falk Eilenberger[4,5], Naga Prathibha Jasti[6,7], David Cahen[7], Oleksandr Kyriienko[8], Christian Schneider[1], Martin Esmann[1,*]

[1]*Institut für Physik, Fakultät V, Carl von Ossietzky Universität Oldenburg, 26129 Oldenburg, Germany*

[2]*Department of Physics, Xiamen University Malaysia, 49300 Sepang, Malaysia*

[3]*University of Applied Sciences Emden/Leer, 26723 Emden, Germany*

[4]*Fraunhofer-Institute for Applied Optics and Precision Engineering IOF, 07745 Jena, Germany*

[5]*Institute of Applied Physics, Abbe Center of Photonics, Friedrich Schiller University, 07745 Jena, Germany*

[6]*Department of Chemistry, Bar-Ilan Univ. Ramat Gan 5290002, Israel*

[7]*Department of Molecular Chemistry and Materials Science, Weizmann Institute of Science, Rehovot 7610001, Israel*

[8]*School of Mathematical and Physical Sciences, University of Sheffield, Sheffield, S10 2TN United Kingdom*

[+]*Corresponding author. Email: m.esmann@uni-oldenburg.de*

## S1: Perovskite synthesis

The crystals were grown as follows below and characterized as described in Refs.[1,2]. In short, butylammonium, $C_4H_9NH_3$ (BA) methylammonium, $CH_3NH_3$ (MA) lead iodide ($BA_2MA_2Pb_3I_{10}$, $C_4N_3$) was crystallized using the slow-cooling method with minor modifications[3]. 5.045 mmol (1.126 g) PbO (ACS reagent, 99.0%, Sigma-Aldrich) was dissolved in 5mL HI (57% in $H_2O$, Sigma-Aldrich) and 850 µL hypophosphorous acid solution (50 wt. % in $H_2O$, Sigma-Aldrich) in an 18 mL vial. After tightly screwing the vial's cap, the mixture was stirred (magnetic stirrer) and heated on a hot plate that was set to 110 °C. The color of the mixture changed from black to clear yellow within a minute. The stirring and heating continued until full dissolution of the PbO (1-2 hours). In the meantime, in an ice-bath, 3 mL of cooled HI (at 4 °C) were mixed with 98 µL butylamine (99.5%, Sigma-Aldrich) by vigorously stirring the HI with a magnetic stirrer and adding the butylamine dropwise. 0.477 g methylammonium iodide (MAI, GreatCellSolar materials), were added to the HI prior to the butylamine addition. The vial was then tightly sealed and stirring continued until no vapor was seen in the upper part of the vial. Once the two mixture was ready, the HI+butylamine+MAI mixture was added, dropwise, to the Pb-containing vial while continuing to stir and heat. This led to the formation of a dark red powder in the vial. Then the vial was tightly sealed and heated, while stirring, until the dark red powder fully dissolved (the hotplate was set for this purpose to 140-170°C for 10-20 minutes). Once the solution was perfectly clear, we carefully took the magnet out of the vial, tightly sealed it again, and transferred into an oven for controlled slow cooling, which was preheated to 105°C. The temperature of the oven was gradually decreased to RT at a rate of 1°C/h. Once the cooling process was completed, large black plates of $BA_2MA_2Pb_3I_{10}$ were seen in the bottom of the vial. The single crystals were then

taken out by evacuating the supernatant and drying them gently with filter paper. To further remove any traces of the solvents, the crystals were then dried in the vacuum oven at 40°C for 12 hours. Structural, optical and electronic characterizations of the crystals used here can be found in Refs.[1,2].

$(BA)_2(MA)_2Pb_3I_{10}$ single crystals were micromechanically exfoliated with the scotch tape method followed by PDMS dry stamping. We first deposit a 10 nm thick hBN bottom layer (2D Semiconductors, substrate at 100°C, 5 min contact time), followed by the quasi-2D HaP flake (substrate at 20°C, 20 min contact, PDMS GelPak grade zero, pre-baked at 85°C for 4h) and another 10 nm thick hBN flake to fully encapsulate the flake (substrate at 20°C, 20 min contact). The preparation was performed under yellow light and finished samples stored in the dark under $N_2$ atmosphere. Figure S1 shows an optical microscope image of a fully-encapsulated quasi-2D HaP flake, deposited on the bottom DBR of the open microcavity. Dark red parts are the perovskite, grey parts are the encapsulating hBN flakes.

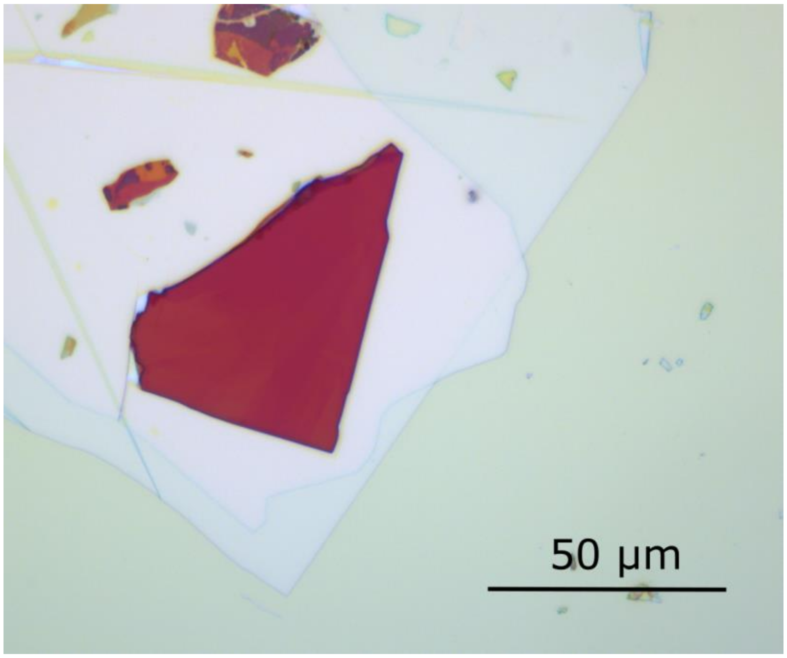


Figure S1: Optical microscope image of a fully encapsulated quasi-2D HaP flake, deposited on the bottom DBR of the open microcavity. Dark red parts are the perovskite, grey parts are the encapsulating hBN flakes.

## S2: Transfer matrix simulations of white light reflectivity

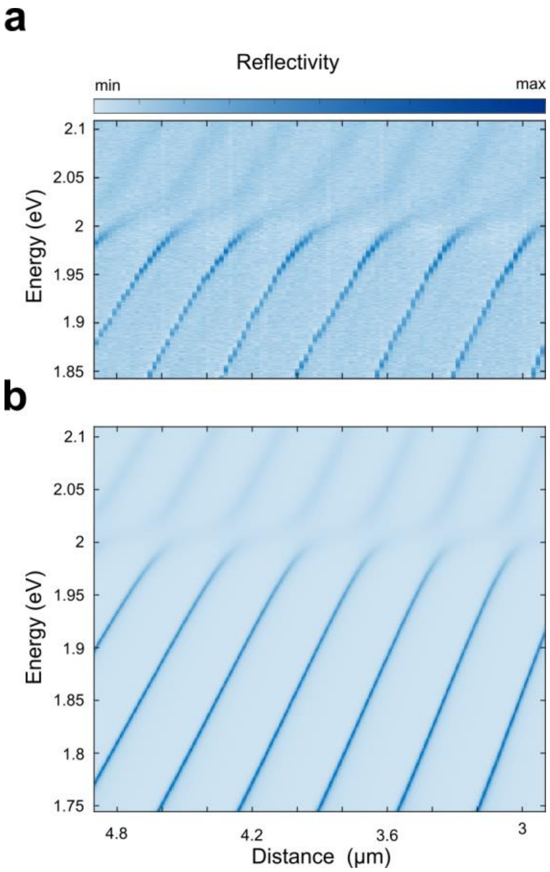


Figure S2: **a** White light reflectivity (WL) spectra as a function of the cavity air gap at normal incidence ($k_{\|} = 0$). Same data as in Fig. 1c of the main text, but with extended distance and energy range. **b** Corresponding transfer matrix simulation with extended energy range. We use this simulation to deduce accurate dispersions for the bare photonic modes in the coupled oscillator model by re-running the simulation with the exciton resonance in the dielectric function of the quasi-2D HaP switched off.

## S3: Description of the coupled oscillator model

To model the observed polariton dispersion in Fig. 1c of the main text, we diagonalize a 5x5 coupled oscillator model based on the coupling Hamiltonian defined in Eq. (S1).

$$H = \begin{pmatrix} E_X & g & g & g & g \\ g & E_{\text{cav},1} & 0 & 0 & 0 \\ g & 0 & E_{\text{cav},2} & 0 & 0 \\ g & 0 & 0 & E_{\text{cav},3} & 0 \\ g & 0 & 0 & 0 & E_{\text{cav},4} \end{pmatrix} \qquad \text{(S1)}$$

Here, $E_X = 2\ \mathrm{eV}$ denotes the exciton energy of the $n = 3$ layered halide perovskite (HaP), $g = 23.5\ \mathrm{meV}$ is the light-matter coupling energy resulting in a normal-mode (Rabi) splitting of $\hbar\Omega = 2g$ in the simplest two-oscillator picture. To obtain accurate values for the uncoupled cavity dispersions $E_{\text{cav},i}(d, k_{\parallel})$ as a function of cavity air gap $d$ and in-plane momentum $k_{\parallel}$, we run transfer matrix simulations of the planar cavity system with the exciton resonance of the HaP switched off (cf. Supplementary Section S2). We find that a simple two-oscillator model is insufficient to describe our experimental observations due to the large normal mode splitting in comparison to the free spectral range of our microcavity and four photonic modes are required for the model to be accurate.

## S4: Angle-resolved photoluminescence

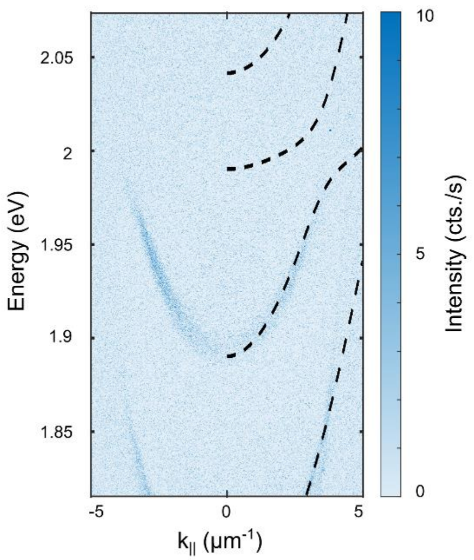


Figure S3: Angle-resolved emission spectrum from planar portion of the DBR cavity shown in Fig. 1 of the main text with an air gap of 3.23 μm under CW excitation at 532 nm. The mode bounding 2 eV from below exhibits all features of a lower polariton branch as a result of an anti-crossing with the $n = 3$ HaP exciton: An inversion point at $k_{\parallel} = 2.5\ \mu\mathrm{m}^{-1}$ and the associated characteristic reduction in group velocity towards higher in-plane momenta. A 5x5 coupled oscillator model (dashed lines) from which we deduce an exciton-photon coupling strength of $g = 23.5\ \mathrm{meV}$, accounts for the observed dispersion relation. No anti-crossing occurs with the exciton associated to the $n = 4$ HaP at 1.9 eV. This exciton functions as an intra-cavity pump for the polariton experiments under strong optical driving.

## S5: Density of electron-hole pairs at the condensation threshold

At the polariton lasing threshold, the excitation conditions described in Fig. 2 of the main text (525 nm central wavelength, 140 fs pulse duration, 80 MHz repetition rate) result in an average number of $N_{\mathrm{ph}} = 6.76\ \mathrm{fJ}/2.36\ \mathrm{eV} \approx 18{,}000$ photons reaching the sample surface per pulse.

By the same kind of transfer matrix simulations as shown Fig. S2, we determine that excitation light with in-plane momenta $\left|\mathbf{k}_{\parallel}\right|/|\mathbf{k}| < 0.3$ is efficiently coupled into the cavity through the first Bragg minimum of the DBRs while larger momenta are reflected. Note that we have chosen our excitation wavelength such that it coincides exactly with the first Bragg minimum of the cavity DBRs at $k_{\parallel} = 0$. The spectral position of the Bragg minimum is largely independent of the length of the cavity air gap. Comparing the effective acceptance NA of 0.3 of the cavity to the used microscope objective with NA=0.65, we obtain an estimated coupling efficiency of 20%. Note that our excitation laser is matched in diameter to the full back aperture of the microscope objective. We furthermore find that the coupled portion of light is absorbed with 66% efficiency for the 218 nm thick HaP flake used in the experiments. Together, these result in an average number of $N_X \approx 3{,}000$ excitons generated per pulse.

We estimate the number of optically active layers as follows: From the relative contributions to the PL spectrum in Fig. 1b of the main text, we estimate the number of layers with $n = 3$ and $n = 4$ in the sample to be the same (equal mixture of phases). From the transfer matrix simulation of our cavity, we deduce an optical penetration depth of $76\ \mathrm{nm}$ for the pump wavelength into the perovskite sample. Assuming $n = 3$ layers and $n = 4$ layers are uniformly distributed across the penetrated depth with layer thicknesses of $2.6\ \mathrm{nm}$ and $3.4\ \mathrm{nm}$, respectively[3], we obtain $N_{\mathrm{HaP}} \approx 13$ optically active layers. With a spot diameter of $d_{\mathrm{spot}} = 0.82 \cdot 525\ \mathrm{nm}/0.3 \approx 1.44\ \mu\mathrm{m}$, this leads to an estimated exciton density of $\rho_{\mathrm{th}} = 1.2 \cdot 10^{10}\ \mathrm{cm}^{-2}$ at the threshold, i.e. one order of magnitude below the Mott density of the quasi-2D HaP material.

## S6: Temperature-dependent redshift of quasi-2D HaP photoluminescence

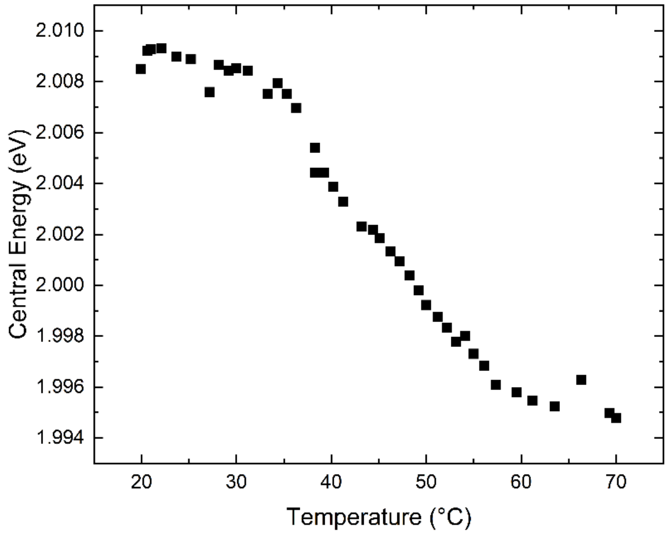


Figure S4: Temperature-dependent PL of an $n = 3$ phase quasi-2D HaP flake deposited on a DBR mirror. The mirror was heated from the back side with a Peltier element, while PL was measured under non-resonant CW excitation at 532 nm. Heating by 40°C from room temperature results in a large redshift by 14 meV. This measurement rules out thermal effects as the origin of the observed blueshift in our system. We find that the redshift was almost completely reversed after cooling down the sample.

## S7: Theoretical model

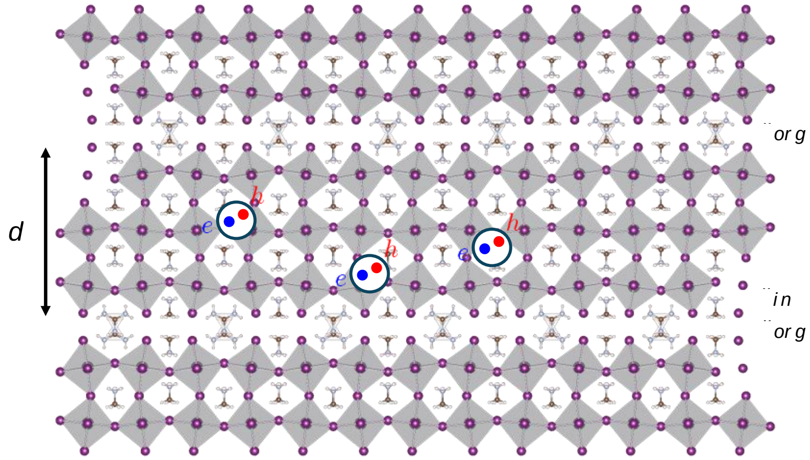


Figure S5: Quantum well structure for theoretical modelling. The exciton bound states (white closed circles) are confined in the inorganic layers (middle region) with length $d$ and dielectric constant $\varepsilon_{\mathrm{in}}$. The inorganic layer is encapsulated by the organic spacers (top and bottom regions) with dielectric constant $\varepsilon_{\mathrm{org}}$.

The properties of electrons and holes in quasi-2D Ruddlesden-Popper perovskites can be approximated to a high precision as a confined motion in a quantum well of length $d$, see Fig. S5. Within this approximation, we can write the exciton wavefunction[4] as

$$X^{+}=\int_{-\frac{d}{2}}^{\frac{d}{2}} dz\, dz' \sum_k \psi(k)\, \phi_e(z)\phi_h(z') a^{+}_{k,z} b_{k,z'}\,,$$

where we separate its in-plane and out-of-plane variables. Here, $\psi(k)$ is the exciton wavefunction with $k$ being the relative in-plane momentum for the electron-hole pair, $\phi_{e/h}(z)=\sqrt{2/d}\cos(\pi z/d)$ is the lowest subband wavefunction for the electrons/holes, with $z$ being their out-of-plane coordinate. The in-plane part can be obtained by solving the Wannier equation[5]

$$\left[\frac{k_e^2}{2m_e}-\frac{k_h^2}{2m_h}\right]\psi(k)-\sum_q V(q)\psi(k+q)=E_b\psi(k)\,,$$

where $m_e/m_0 = 0.097$ and $m_e/m_0 = 0.141$, with $m_0$ being the free electron mass[3], and the interacting kernel is

$$V(q)=\int_{-\frac{d}{2}}^{\frac{d}{2}}\int_{-\frac{d}{2}}^{\frac{d}{2}} dz\, dz' |\phi_e(z)|^2 W(q,z,z')\, |\phi_h(z)|^2\,.$$

The electron and hole interacting potential is obtained by solving the mesoscopic Poisson's equation[6–8], $\varepsilon(q^2+\partial_z^2)W(q,z,z') = e/\varepsilon_0\delta(z-z')$. The corresponding Coulomb potential is

$$W(q,z,z')=\frac{4\pi e^2}{\varepsilon_0}\frac{\cosh[\, q\left(\frac{1}{2}d-z\right)+\eta]\cosh[\, q\left(\frac{1}{2}d+z'\right)+\eta]}{\sinh(qd+2\eta)}$$

with $\eta=\frac{1}{2}\ln\frac{\varepsilon_{\rm in}+\varepsilon_{\rm org}}{\varepsilon_{\rm in}-\varepsilon_{\rm org}}$. The dielectric constant for the inorganic layers and the organic spacer is $\varepsilon_{\rm in}=5.2$ and $\varepsilon_{\rm org}=2.2$ for $n=3$ [9]. Using the Gaussian basis method[4], we obtain the binding energy $E_b = 180$ meV and the wavefunction $\psi(k)$.

In the cavity, the exciton and photon hybridize forming polaritons with the Hamiltonian

$$H_{\rm pol}=\begin{bmatrix}\omega_c & \frac{1}{2}\Omega\\ \frac{1}{2}\Omega & E_X\end{bmatrix},$$

where $\omega_c$ is the cavity photon energy, $\Omega$ is the Rabi splitting, and $E_X$ is the exciton energy. This gives the polariton energies $E_{\pm}=\frac{1}{2}\left[\omega_c+E_X\pm\sqrt{(\omega_c-E_X)^2+\Omega^2}\right]$.

The non-linear blueshift of the lower polariton energy $E_{-}(\rho_X)$ at increasing exciton density $\rho_X$ has two contributions. The first one originates from the Pauli blockade, as excitons are not pure bosons and consist of electrons and holes. The increased occupation reduces the available area (volume) to create excitons, leading to an effective reduction of the light-matter coupling. This in turn reduces the Rabi splitting as

$$\Omega(\rho)=\Omega_0-\rho_X g_s.$$

The corresponding rate of reduction can be estimated from the excitonic wavefunctions calculated for our structure, leading to a rate of $g_s = A\,\Omega_0 \sum_k |\psi(k)|^4 \approx 2.7\ \mu\text{eV}\mu\text{m}^2$. The second contribution comes from the exciton-exciton Coulomb interactions. Here, the dominant contribution is the

exchange between electrons and exchange between holes, leading to a shift of the exciton energy scaling in the lowest order as

$$E_X(\rho_X) = E_0 + \rho_X g_X,$$

where $g_X = 2\sum_{k,k'} V(k-k')[|\psi(k)|^2(|\psi(k')|^2 - \psi^*(k)\psi(k'))] \approx 1\ \mu\mathrm{eV}\mu\mathrm{m}^2$ [10]. Therefore, the blueshift of the lower polaritons at linear order in $\rho$ is

$$E_-(\rho) - E_-(0) \approx \frac{1}{2}\left[\left(1 + \frac{\omega_c - E_0}{\Delta}\right) g_X + \frac{\Omega_0}{\Delta} g_s\right] \rho_X$$

with $\Delta^2 = (\omega_c - E_0)^2 + \Omega_0^2$. From experiments, we have $\omega_c \approx 1.81$ eV for the cavity photonic mode and $E_0 \approx 2$ eV for the excitonic mode. Using the information above, we can estimate the non-linear interaction coefficient as

$$\frac{1}{2}\left[\left(1 + \frac{\omega_c - E_0}{\Delta}\right) g_X + \frac{\Omega_0}{\Delta} g_s\right] \approx 0.34\ \mu\mathrm{eV}\mu\mathrm{m}^2\ ,$$

where we have effectively included the dependence on Hopfield coefficients for the strongly-detuned polaritonic mode. For the non-linear shift in Fig. 2c of the main text, we observe that the experimental estimate for the non-linear constant is $\delta_{\mathrm{th}}/\rho_{\mathrm{th}} \approx 5.4\ \mu\mathrm{eV}\mu\mathrm{m}^2$. This is approximately within an order of magnitude to our theoretical estimate, which is on the conservative side (cf. Section S8 below for comparison to a second set of input-output data on the same quasi-2D HaP flake). In fact, recent results from the diamagnetic coefficient measurements suggest that the Bohr radius of Ruddlesden Popper perovskite excitons is even larger than considered before (possibly due to screening and mass renormalization). This can explain the stronger interaction observed experimentally. Other points to consider include the need of independent density estimates and potential build-up of excitons in the reservoir. As the pump repetition rate is sufficiently high, we may induce a population of dark excitons that do not contribute to PL, but via phase space filling influence the non-linear shifts. We consider that further research in this direction, as well as treatment of disorder physics can help elucidating the nature of polaritonic non-linearity in quasi-2D Ruddlesden Popper perovskite condensates.

## S8: Input-output characteristic, second dataset

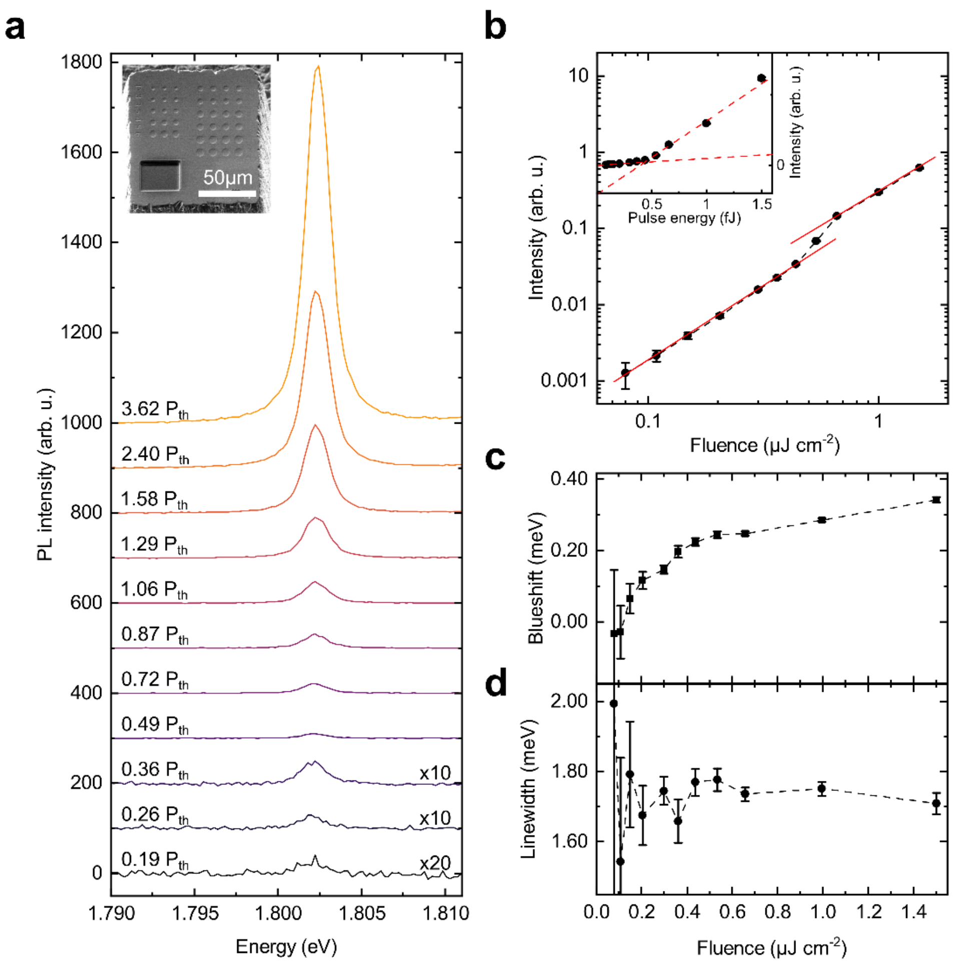


Figure S6: Additional input-output curve acquired on a different section of the same sample as studied in Fig. 2 of the main text. **a** Power-dependent polariton emission spectra as a function of excitation pulse energy **b** Double logarithmic plot of the input-output curve extracted from the areas under Voigt fits to the spectra in a. **c** Spectral position of the Voigt fit as a function of pulse energy relative to the first data point. The emission undergoes a blueshift continuing beyond the lasing threshold. **d** Spectral linewidth extracted from the Voigt fits (see Methods section of the main text) as a function of pulse fluence. The observed threshold fluence is virtually identical to the data in Fig. 2 of the main text, while the blueshift and linewidth reduction in panels c and d are less pronounced. We attribute this to the smaller exciton Hopfield coefficient, since the data were acquired at a slightly more red-detuned lower polariton energy of 1.80 eV. Error bars in b-d correspond to 95% confidence intervals of the fit.

The additional input-output curve in Fig. S6 was acquired on a different section of the same sample studied in Fig. 2 of the main text, however with the lower polariton slightly further red detuned such that the exciton Hopfield coefficient was reduced by a factor of 0.87. We observe virtually identical threshold fluence, while the estimated nonlinear scattering coefficient derived from Fig. S6c amounts to $\delta_{\mathrm{th}}/\rho_{\mathrm{th}} \approx 1.9\ \mu\mathrm{eV}\mu\mathrm{m}^2$, i.e. a factor of 2.5 smaller than in Fig. 2 of the main text. Again, this value is consistent within approximately an order of magnitude agreement with our theoretical estimate in Section S7.

In the simplest case of exciton-exciton scattering the expected polariton blueshift scales quadratically with the exciton fraction, $\Delta \sim |X|^4$. Hence, for otherwise the same conditions the observed variation in nonlinear scattering coefficient between the two datasets should be a factor

of 1.33. However, we note that this is clearly oversimplified and several contributions need to be accounted for when comparing results:

First, as discussed in Section S7, nonlinear shifts originate from the combination of exciton-exciton scattering and nonlinear phase space filling, modifying the scaling, where the saturation-based shift scales as $|X|^{3/2}$ [11]. Second, the absolute value of the nonlinear shift depends on the effective area $A_{\mathrm{eff}}$ that we measure, as well as the structural composition. While performed on the same perovskite flake, the exact location on the flake was different between datasets. It is likely that the vertical composition of the mixed phase crystal thus showed slight differences despite virtually identical PL spectra. This can contribute to the modification of the effective Bohr radius. Also, since the disorder landscape may vary between locations on the flake, we may expect changes of $A_{\mathrm{eff}}$ at the level of tens of percent.

We, however, note that despite these possibilities the observed polariton blueshift in both measurements agrees with the theoretical estimate to approximately one order of magnitude. This level of agreement is rather common, even when one compares exciton-polariton nonlinearities reported in III-V materials[12]. We stress that our study provides a solid indicative range of nonlinear scattering coefficients found for the quasi-2D HaP system under investigation.

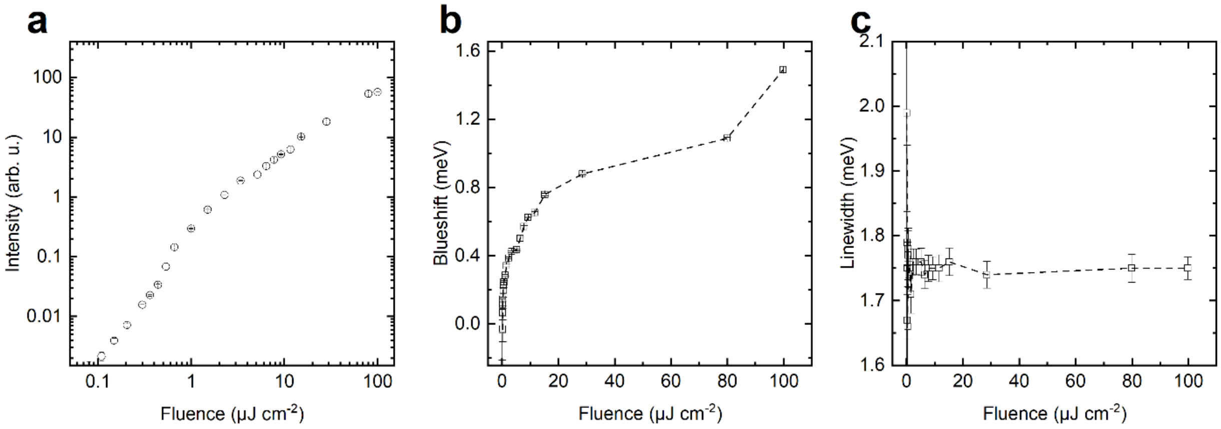


Figure S7: **a** Double logarithmic plot of the input-output curve in Fig. S6b with fluence range extended up to $P = 240P_{th}$. **b** Spectral position of the polariton emission as a function of pulse fluence relative to the first data point. The emission undergoes a blueshift continuing beyond the lasing threshold with indications of an accelerated shift at very large pulse fluences. **c** Spectral linewidth of the emission as a function of pulse fluence. Error bars correspond to 95% confidence intervals of the Voigt fit.

## S9: Optical setup

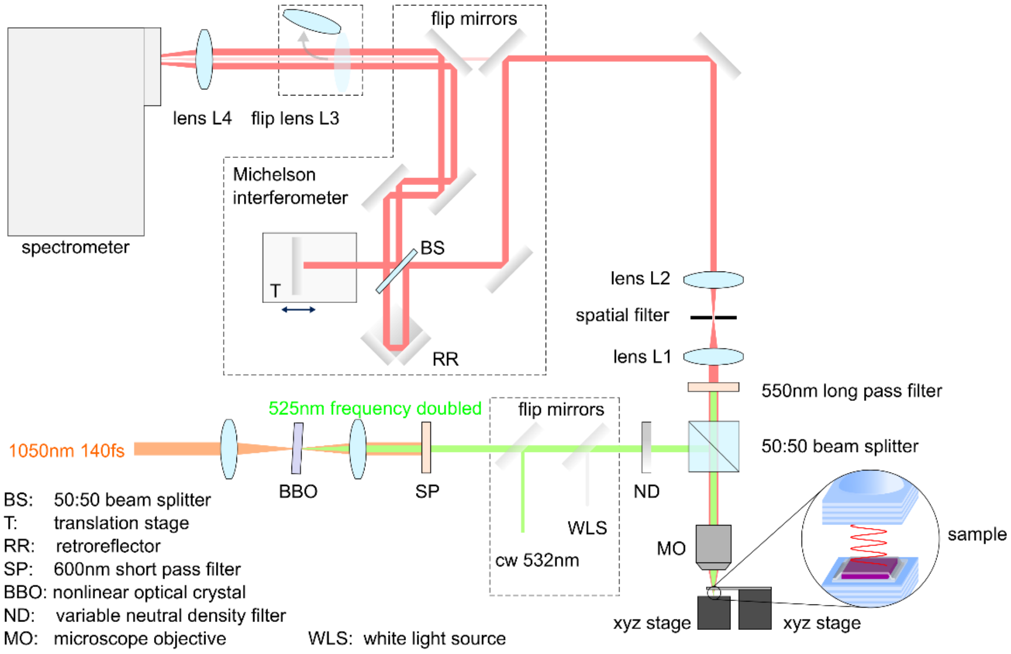


Figure S8: Schematic of the experimental setup used to measure the spatial coherence of the polariton condensate.

**Excitation path**: Femtosecond pulses from a mode locked Ti:Sapphire laser source (Chameleon, Coherent) operated at 1050 nm with 80 MHz repetition rate, are focused on a non-linear optical crystal (BBO, Crysmit optics, 21.3° cut, 0.1 mm thickness) to generate frequency doubled pulses at 525 nm. The light from the BBO is collected and collimated by a lens, before a 600 nm short pass filter isolates the frequency doubled light. For white light reflection or linear photoluminescence measurements, light from a white light source (Thorlabs SLS301) or from a continuous wave DPSS laser (532 nm) can be coupled in the excitation path by flip mirrors (dashed box). The excitation power can be controlled with a motorized variable neutral density filter. The excitation light is reflected by a 50:50 beam splitter towards a microscope objective (50X Mitutoyo Plan Apo NIR HR 0.65NA), which focuses the light on the sample. For polariton experiments, the active material is prepared in an open cavity configuration as described in [13]. Dispersion by the beam splitter and the microscope objective lead to a pulse length of the frequency doubled pulses of about 140 fs.

**Collection path**: Operating in reflection geometry, the same microscope objective collects the emission from the sample (microcavity) and transmits it through the beam splitter. Reflected excitation laser light is filtered out by a 550 nm long pass filter. Lens L1 with its focal point positioned on the back aperture of the microscope objective focuses the emission to a spatial filter, where lens L2 in confocal configuration collimates the light again. Lens L4 images the emission onto the entrance slit of an imaging spectrometer (Andor Shamrock 500i), with a Peltier-cooled EMCCD camera (Andor iXon Ultra 888, operated without EM gain). Adding flip lens L3 (top left dashed box) in a Fourier imaging configuration allows for angle-resolved measurements,

by imaging the back-focal plane of the microscope objective onto the entrance slit of the spectrometer.

**Michelson interferometer**: Additionally, a Michelson interferometer can be added to the collection bath by two flip mirrors (large dashed box). The beam is split by a 50:50 beam splitter (BSW10R), with the reflected part again being reflected back to the beam splitter by a retroreflector (PS976M-B) generating a spatial offset to the transmitted beam, that is reflected back to the beam splitter by a planar mirror on a motorized translation stage (PI Linear Stage M-511). The retroreflector introduces a point reflection with respect to the beam reflected by the planar mirror. Varying the position of the translation stage adds a temporal offset between the beams. Furthermore, the spatial offset between the two beams creates a difference in wave vectors between the two beams when focused onto the spectrometer slit by lens L4. This creates a time delay-dependent fringe pattern on the CCD camera, which is used to extract information about the spatial coherence.

### S10: White light reflectivity and Photoluminescence of a mixed phase perovskite

To precisely determine the exciton energies, we exfoliated another quasi-2D HaP flake from the same bulk crystal that was used for the samples described in the main text. The crystal has contributions from both $n = 4$ and $n = 3$ phases. In Fig. S9, we show a photoluminescence (PL, blue) and a white light (WL, red) reflectivity spectrum of the flake after it was transferred onto a DBR with identical properties compared to the DBRs used for the measurements in the main text. The PL shows two distinct peaks at 1.995 eV and 1.92 eV respectively, corresponding to the $n = 3$ and $n = 4$ phase exciton of the crystal. The WL reflectivity spectrum shows a dip at 1.99 eV, corresponding to the $n = 3$ exciton and a significantly shallower absorption dip at ~1.93 eV, corresponding to the $n = 4$ exciton.

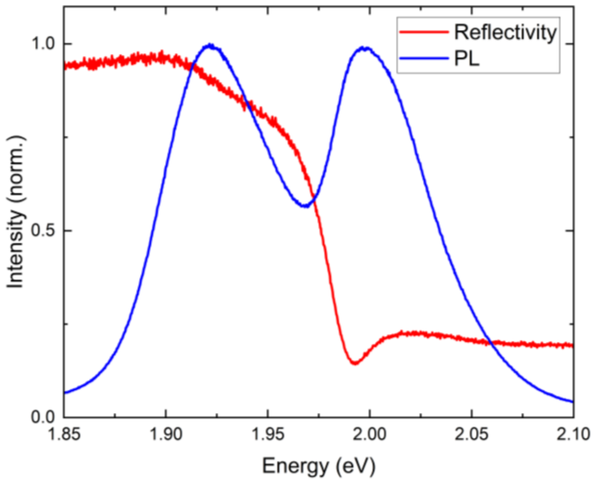


Figure S9: Photoluminescence (PL, blue) and white light (WL, red) reflectivity spectrum of a mixed phase layered perovskite flake derived from the same bulk crystal used for the polariton condensation experiments. Two resonances corresponding to the $n = 3$ and the $n = 4$ exciton are clearly visible in both PL and WL reflectivity.

A strong coupling analysis for the particular flake investigated in Fig. S9 is shown in Fig. S10 below. At 2 eV, a Rabi splitting associated with the $n = 3$ phase quasi-2D HaP exciton is observed, with a coupling strength of 16 meV. The reduced coupling strength compared to the data in Fig. 1 of the main text is attributed to the reduced flake thickness of 200 nm. Importantly, no evidence of a Rabi gap is observed at 1.92 eV, the energy of the $n = 4$ phase quasi-2D HaP exciton. Instead, the data reveals a clear dispersive behavior attributed to the variation in the real part of the refractive index. This is in agreement with the much lower absorption for $n = 4$ shown in Fig. S9. We note that the dispersive feature is almost absent in the device discussed in the main text, which hints at an even lower contribution from the $n = 4$ phase. These results lead to the conclusion that the $n = 4$ HaP phase does not couple strongly to the cavity photon and can hence act as an effective intra-cavity pump as discussed in the main text and supported by the absence of strong coupling features around 1.92 eV in the PL dispersion shown in Fig. S3.

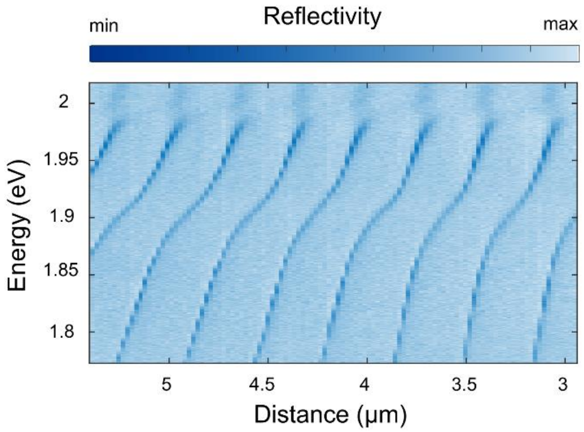


Figure S10: Strong coupling measurement of a layered perovskite flake with a thickness of 200 nm. At 2 eV, the $n = 3$ exciton shows strong coupling with the cavity photon, the $n = 4$ exciton at 1.92 eV only results in a dispersive feature and no Rabi gap. The dispersive feature is almost absent in the device discussed in the main text, for which full strong coupling data are shown in Fig. S2. This hints at an even lower $n = 4$ contribution for the device used for polariton condensation experiments and is in full agreement with the absence of a Rabi gap at 1.92 eV in the PL data presented in Fig. S1 of the Supplementary Information.

In an effective medium picture, i.e. when describing the mixed phase quasi-2D HaP by a weighted average of the complex refractive indices for $n = 3$ and $n = 4$ phase, the reason why the $n = 4$ phase couples weakly in the present scenario, becomes evident: While pure phase $n = 3$, as well as pure phase $n = 4$ perovskite, may both enter the strong coupling regime and anti-cross with photonic cavity modes, the $n = 4$ exciton loses oscillator strength via the averaging of the two refractive indices. This results in a transition to the weak coupling regime. Since the $n = 3$ exciton transition occurs at a higher energy, averaging with the complex refractive index of $n = 4$ results in persistent contributions of the $n = 4$ Lorentz oscillator to the oscillator strength of the $n = 3$ resonance. These contributions are strong enough to retain the strong coupling conditions for $n = 3$.

## S11: Input-output characteristic, pure n=3 phase perovskite

To clarify the role of the $n = 4$ phase in our experiments, we ran polariton lasing studies on a pure $n = 3$ phase perovskite crystal under otherwise equal conditions to the experiment reported in Fig. 2 of the main text. The results are shown below in Figs. S11 and S12. Fig. S11 shows white light reflectivity (WL) and photoluminescence (PL) spectra of a pure $n = 3$ phase $(BA)_2(MA)_2Pb_3I_{10}$ flake that was prepared following the same procedure as outlined in the methods section. Comparing these spectra to Fig. S9 above (PL and WL for mixed phase crystal), the $n = 3$ contributions are clearly visible in WL as an absorptive feature at 2 eV and a single peak in PL at roughly the same energy. The features attributed to $n = 4$ (shallow absorption in WL and second peak in PL at 1.93 eV) are absent.

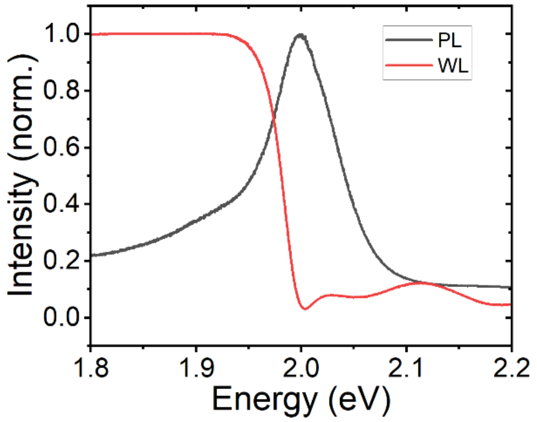


Figure S11: Photoluminescence (PL, black) and white light (WL, red) reflectivity spectrum of a pure $n = 3$ phase layered perovskite flake derived from the same bulk crystal used for the polariton condensation experiments. The resonance corresponding to the $n = 3$ exciton is clearly visible in both PL and WL reflectivity.

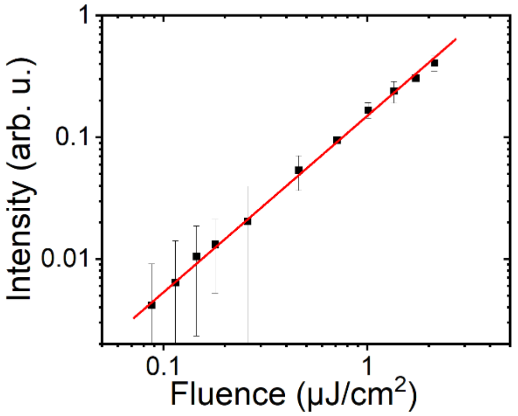


Figure S12: Input-output curve acquired on the pure $n = 3$ sample characterized in Fig. S11. Double logarithmic plot of the input-output curve extracted from the areas under Voigt fits to the emission spectra (analogous procedure to the data analysis for the mixed phase crystal presented in Fig. 2 of the main text). The same excitation conditions and the same sphere-cap upper mirror of the cavity were used. For the same range of input fluences as shown in Fig. 2 no sign of an optical non-linearity is observed. Error bars correspond to 95% confidence intervals of the fit.

We performed polariton lasing experiments on this new sample following the exact same procedure used for the mixed phase perovskite in Fig. 2 of the main text. The flake was fully hBN encapsulated and placed on a DBR mirror equal to the ones used in the main text. We completed the open cavity with the same sphere cap shaped indentation used before.

Input-fluence dependent PL spectra were evaluated in the same way as for Fig. 2 of the main text. The resulting input-output curve showing the area under a Voigt-fit to the PL spectrum as a function of input fluence in double logarithmic representation is shown in Fig. S12. We observe a linear behavior with no sign of optical non-linearity.

From these results we conclude that the presence of the $n = 4$ contribution in our mixed phase crystals is instrumental in observing polariton lasing in our case. The most likely scenario for the origin of this effect is the intra-cavity pumping outlined in our manuscript.

### S12: Input-output characteristic, planar cavity

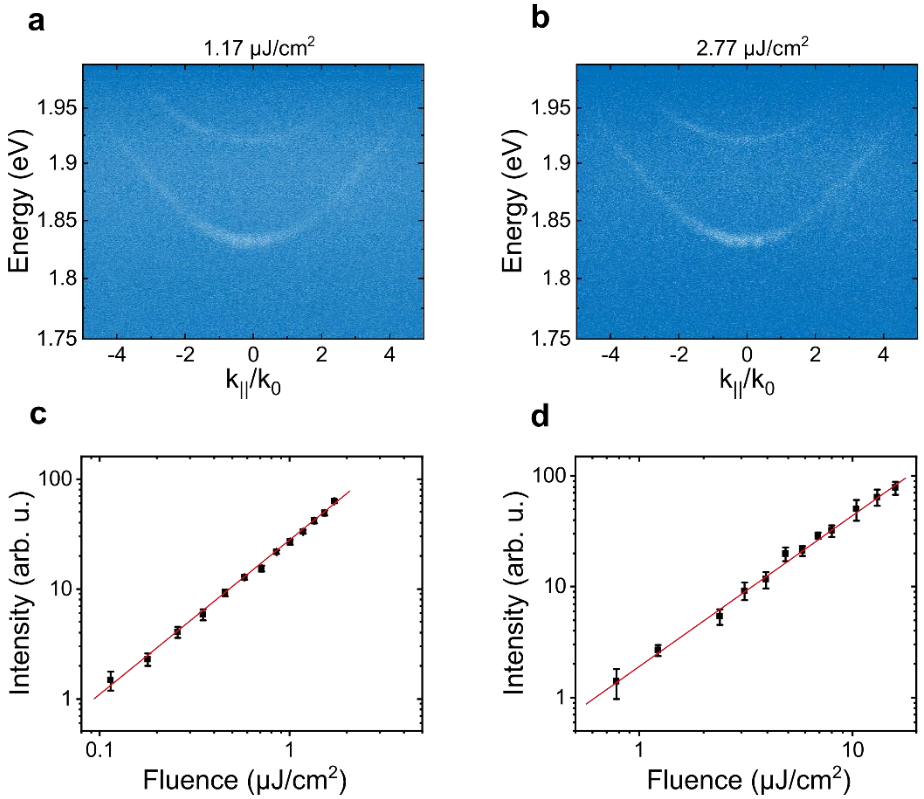


Figure S13: **a**,**b**: momentum-resolved PL under pulsed non-resonant excitation for the same mixed phase perovskite flake as used in the dataset for Fig. S9/10 of the supplementary information. The detuning and upper mirror were the same as in the main text, but we used a planar section of the upper mirror. For fluences slightly (a) and well above (b) the polariton condensation threshold reported in the main text, no distinct change in PL distribution towards a macroscopic occupation around $k_{\|} = 0$ is observed. **c**,**d**: Input-output curve acquired on the sample, fine scan for low fluences in c, coarser scan up to large fluences in d. Error bars in c-d correspond to 95% confidence intervals of the fit.

To see whether the 0D confinement is instrumental in reaching the polariton condensation regime in our system, we have performed additional input-output studies on planar sections of the microcavity. The results of these measurements are shown in Fig. S13. Panels (a) and (b) show momentum-resolved PL under pulsed non-resonant excitation for the same mixed phase perovskite flake as used in the dataset for Fig. S9/10. The detuning and upper mirror were the same as in the main text, but we used a planar section of the upper mirror. For fluences slightly above (a) and well above (b) the polariton condensation threshold reported in the main text, no distinct change in PL distribution towards a macroscopic occupation around $k_{\|} = 0$ is observed.

We furthermore recorded detailed input-output characteristics of the planar cavity device. The results are presented in panel (c) showing a fine scan for small pump fluences in the fluence range for which the polariton condensation threshold was observed with 0D confinement and in panel (d) showing a coarser scan over a wider range of pump fluences reaching up to $35P_{\mathrm{th}}$ with

$P_{\mathrm{th}} = 0.41\ \mu\mathrm{J/cm}^2$ the threshold observed with 0D confinement under otherwise equal conditions on the same perovskite flake. Both input-output curves in panels (c) and (d) show no deviation from a linear trend underpinning that 0D confinement is indeed largely helpful for reaching polariton condensation at very moderate pump fluences.

We note that the enhancement of stimulated scattering and the trend towards lower polariton lasing thresholds under 0D confinement is well established in the literature. The enhancement of stimulated scattering in confined polariton system was discussed in detail in [14]. The phenomenon was for example exploited for demonstrations of polariton lasing in monolithic III-V semiconductor microcavities where 0D confinement was established by laterally etching the microcavity into micropillars [15].

**Supplementary References**


1. Aharon, S. *et al.* 2D Pb-Halide Perovskites Can Self-Heal Photodamage Better than 3D Ones. *Adv. Funct. Mater.* **32**, 2113354 (2022).
2. Jasti, N. P. *et al.* Experimental evidence for defect tolerance in Pb-halide perovskites. *Proc. Natl. Acad. Sci.* **121**, e2316867121 (2024).
3. Stoumpos, C. C. *et al.* Ruddlesden–Popper Hybrid Lead Iodide Perovskite 2D Homologous Semiconductors. *Chem. Mater.* **28**, 2852–2867 (2016).
4. Ceferino, A., Song, K. W., Magorrian, S. J., Zólyomi, V. & Fal'ko, V. I. Crossover from weakly indirect to direct excitons in atomically thin films of InSe. *Phys. Rev. B* **101**, 245432 (2020).
5. Latini, S., Olsen, T. & Thygesen, K. S. Excitons in van der Waals heterostructures: The important role of dielectric screening. *Phys. Rev. B* **92**, 245123 (2015).
6. Keldysh, L. V. Coulomb interaction in thin semiconductor and semimetal films. *Jetp Lett.* **29**, 658 (1979).
7. Rytova, N. S. The screened potential of a point charge in a thin film. *Mosc. Univ. Phys. Bull.* **3**, 18 (1967).

8. Trolle, M. L., Pedersen, T. G. & Véniard, V. Model dielectric function for 2D semiconductors including substrate screening. *Sci. Rep.* **7**, 39844 (2017).
9. Blancon, J.-C. *et al.* Scaling law for excitons in 2D perovskite quantum wells. *Nat. Commun.* **9**, 2254 (2018).
10. Combescot, M., Betbeder-Matibet, O. & Dubin, F. The many-body physics of composite bosons. *Phys. Rep.* **463**, 215–320 (2008).
11. Betzold, S. *et al.* Coherence and Interaction in Confined Room-Temperature Polariton Condensates with Frenkel Excitons. *ACS Photonics* **7**, 384–392 (2020).
12. Estrecho, E. *et al.* Direct measurement of polariton-polariton interaction strength in the Thomas-Fermi regime of exciton-polariton condensation. *Phys. Rev. B* **100**, 035306 (2019).
13. Lackner, L. *et al.* Tunable exciton-polaritons emerging from WS2 monolayer excitons in a photonic lattice at room temperature. *Nat. Commun.* **12**, 4933 (2021).
14. Paraïso, T. K. *et al.* Enhancement of microcavity polariton relaxation under confinement. *Phys. Rev. B* **79**, 045319 (2009).
15. Bajoni, D. *et al.* Optical Bistability in a GaAs-Based Polariton Diode. *Phys. Rev. Lett.* **101**, 266402 (2008).
